# Fermented black radish (*Raphanus sativus* L. var. *niger*) attenuates methionine and choline deficient diet‐induced nonalcoholic fatty liver disease in mice

**DOI:** 10.1002/fsn3.1200

**Published:** 2019-09-09

**Authors:** Meejung Ahn, Jeongtae Kim, Yuna Choi, Poornima Ekanayake, Ji‐Yeon Chun, DaWun Yang, Gi-Ok Kim, Taekyun Shin

**Affiliations:** ^1^ Department of Veterinary Anatomy College of Veterinary Medicine and Veterinary Medical Research Institute Jeju National University Jeju Republic of Korea; ^2^ Department of Food Bioengineering College of Engineering Jeju National University Jeju Republic of Korea; ^3^ Jeju Biodiversity Research Institute Jeju Technopark Seoguipo Republic of Korea

**Keywords:** fermented black radish, fibrosis, inflammation, MCD diet, steatosis

## Abstract

As one of the wide‐ranging form of chronic liver disease, there are only limited therapeutic options for nonalcoholic fatty liver disease (NAFLD). We evaluated whether fermented black radish (*Raphanus sativus* L. var. *niger*; FBR) ameliorates lipid accumulation, inflammation, and hepatic fibrosis, which are characteristics of the pathogenesis of NAFLD. Fermented black radish treatment reduced lipid accumulation in 3T3‐L1 adipocytes, which appeared to be associated with the downregulation of adipogenic transcription factors, including sterol regulatory element‐binding protein 1c, CCAAT/enhancer‐binding protein α, peroxisome proliferator‐activated receptor γ, and lipid accumulation‐related genes including adipocyte protein‐2 and fatty acid synthase. Administration of FBR to C57BL/6J mice challenged with methionine and choline deficient (MCD) diet significantly attenuated the increased serum levels of alanine aminotransferase, aspartate aminotransferase, alkaline phosphatase, and triglyceride. In addition, treatment with FBR interestingly repressed the hepatic inflammation induced with MCD diet, by lowering the expression of inducible nitric oxide synthase and suppressing the inactivation of macrophages and Kupffer cells in the liver. Fermented black radish was also shown to mitigate liver fibrosis through the inhibition of alpha‐smooth muscle actin, transforming growth factor beta‐1, and collagen type I alpha 1 chain. Our results indicate that FBR ameliorates NAFLD and its related metabolic disease by regulating multiple pathways, suggesting that FBR may be an effective dietary supplement for ameliorating NAFLD.

## 1 INTRODUCTION

Nonalcoholic fatty liver disease (NAFLD) is a clinical‐pathological syndrome that is described as fatty infiltration of the liver exceeding 5%–10% by weight. As the name itself implies, this particular liver pathology is not associated with alcohol intake and exhibited by hepatic steatosis (Lee, Nam, Seong, & Ryu, 2018) and adipogenesis (Ma et al., 2012). 3T3‐L1 adipocytes and a methionine and choline deficient (MCD) diet are a widespread cell culture model (Li et al., 2017) and dietary animal models of NAFLD (Lee et al., 2018), respectively. Choline and methionine are two essential nutrient components deficiency of which impede beta‐oxidation and synthesis of very low‐density lipoprotein (VLDL) particles (Ibrahim, Hirsova, Malhi, & Gores, 2016). Additionally, choline deficiency impairs hepatic secretion of VLDL, leading to hepatic pathophysiological changes, as well as inflammation and fibrosis (Lau, Zhang, & Yu, 2017). The MCD diet‐induced model of liver injury is characterized by elevated triglyceride (TG) and free fatty acid levels in the liver, as well as lowered serum TG levels (Lee et al., 2018). Hepatocellular damage in this model as described with histopathology include hepatocyte ballooning and cell death, along with infiltration of inflammatory cells or varying degrees of fibrosis (Yeh & Brunt, 2014).

Black radish (*Raphanus sativus* L. var. *niger*), a subtype of radish (*Raphanus sativus* L.), is an edible root that exhibits diverse biological effects (Ahn et al., 2018). Black radish contains a high concentration of total glucosinolate, an important component (Ciska, Martyniak‐Przybyszewska, & Kozlowska, 2000). Previous studies have shown that black radish extract provides hepatoprotective effects on carbon tetrachloride‐induced hepatic injury (Ahn et al., 2018). Black radish also represses the membrane changes caused by a high‐fat diet while enhancing the natural scavenging activity protecting mucosal membranes against lipid peroxidation in rat colon (Sipos, Hagymasi, Lugasi, Feher, & Blazovics, 2002). Recently, fermentation by microorganisms such as *Lactobacillus* spp*.* has been used to disassociate fibers in vegetables and to release bioactive substances, leading to health benefits (Patra, Das, Paramithiotis, & Shin, 2016). Previously we reported that fermented black radish (FBR) ameliorates carbon tetrachloride‐induced acute hepatic injury through antioxidant and radical scavenging effects (Kim et al., 2017). Nonetheless, only little is known with respect to the effects of FBR on MCD diet‐induced hepatic injury.

We investigated the effects of FBR in mice with MCD diet‐induced chronic liver injury, and to examine the potential mechanisms of protective effects with respect to steatosis, inflammation, and fibrosis of the liver.

## 2 MATERIALS AND METHODS

### 2.1 Preparation of FBR

A reorganized method of vegetable fermentation was applied. Black radish was acquired from a local farm in Jeju‐do, Korea, and cleaned and sterilized for 15 min at 95°C. This was followed by grinding to obtain fine particles. The resulting particles were mixed with distilled water (1:1), and the suspension was autoclaved for 15 min at 121°C. The black radish suspension was then inoculated with *Lactobacillus plantarum* (Korean Culture Center of Microorganisms, KCCM) at a concentration of 0.7%–1.0% and incubated in De Man, Rogosa, and Sharpe (MRS) agar for 24 hr at 37°C, then propagated in MRS broth under the same conditions. After fermentation for 48 hr in a shaking incubator, cessation of fermentation was achieved by heating at 95°C for 15 min. Finally, FBR was freeze‐dried and further ground to approximately 15% (w/w) and packed in vacuum aluminum foil bags to be stored at 4°C until further use.

### 2.2 Cell culture and adipocyte differentiation

The cells were cultured in DMEM (Gibco) supplemented with 10% fetal bovine serum (FBS; Gibco) and 1% penicillin‐streptomycin (Gibco) in a 37°C incubator at 5% CO_2_. The adipocytes were induced with 0.5 mM 3‐isobutyl‐1‐methylxanthine (Sigma‐Aldrich), 1 μM dexamethasone (Sigma‐Aldrich), and 10 μg/ml insulin (Sigma‐Aldrich). Differentiated adipocytes were treated with FBR and then cultured for 6 days.

### 2.3 Oil Red O staining

To reveal lipid droplets, 3T3‐L1 cells and fixed MCD diet‐induced liver tissue were stained with Oil Red O assay. The samples were stained for 20 min with 0.6% Oil Red O solution at room temperature to allow stained lipid droplets to form. After staining, the cells were visualized using a microscope (Olympus BX53; Olympus Corp.). After eluting of the Oil Red O dye, the absorbance at 490 nm was determined by microplate leader.

### 2.4 Determination of triglyceride contents in cells

Mouse 3T3‐L1 cells were extracted with 5% NP‐40 for the preparation of whole‐cell proteins. Measurement of the TG level in 3T3‐L1 cells extracts was carried out using TG assay kits (Abcam).

### 2.5 Animals

C57BL/6J mice (male, 7 weeks old; Central Laboratory Animal Inc.) were used in the present study. Animals were housed at a controlled temperature of 24 ± 2°C with 12‐hr light/dark cycles and supplied with a standard diet and water ad libitum. All experiments were performed in accordance with protocols approved by the Institutional Animal Care and Use Committee (IACUC) of the Korea Institute of Science and Technology and Jeju National University (permit number: 2016‐0040).

**Figure 1:**
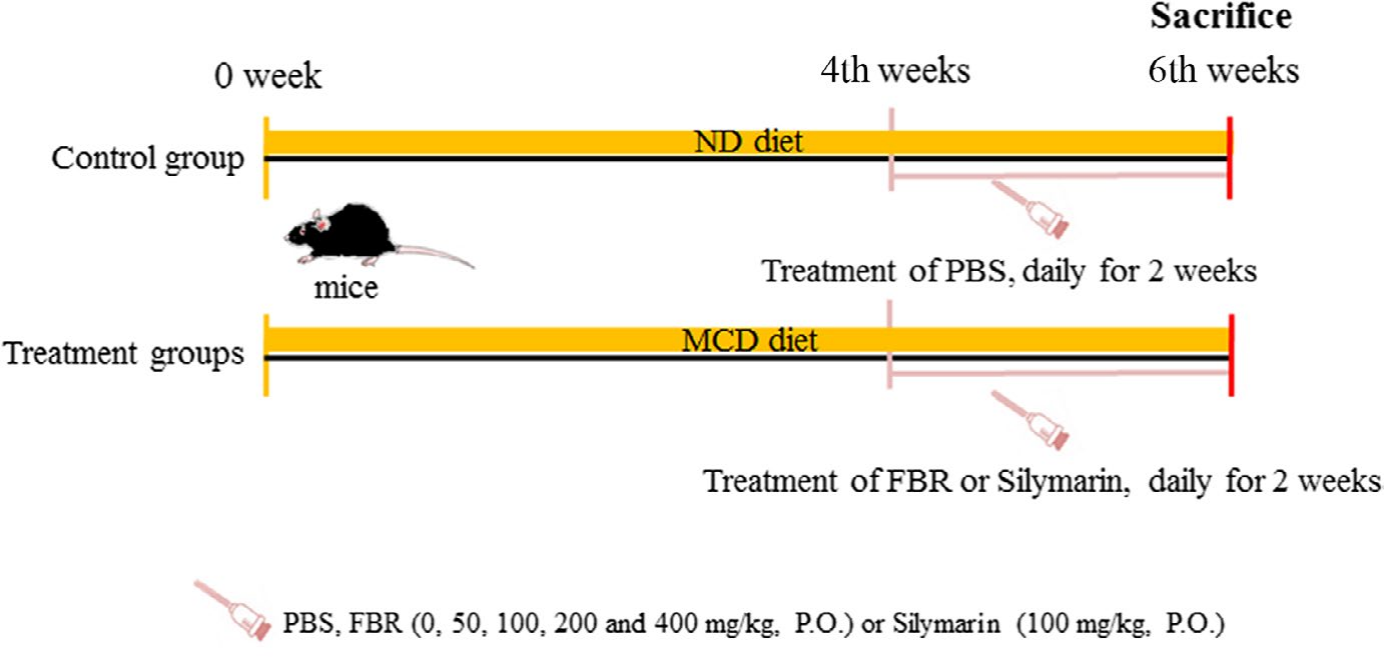
Schematic drawing of the in vivo experimental schedule

A mouse model of NAFLD was established using the MCD diet (Research Diets, Inc.). Mice in the control group were fed a normal chow diet (Central Laboratory Animal Inc.), whereas mice in the other groups were fed the MCD diet. While establishing the NAFLD model over a period of 6 weeks, the mice in each treatment group were administered phosphate‐buffered saline (PBS), FBR, and silymarin (Sigma‐Aldrich, S0292, Lot #BCBP0730V) as a positive control once daily for 2 weeks by oral gavage. Experimental animals were randomly divided into seven groups (*n* = 10 per group): normal control (normal chow diet), vehicle‐treated (MCD + PBS), positive control (MCD + 100 mg/kg silymarin), and four test groups (MCD + FBR). In the four test groups, FBR was administered at doses of 50, 100, 200, and 400 mg/kg for 2 weeks after 4 weeks of the MCD diet (Figure 1). At the time of sacrifice, blood samples and liver tissues were collected for serum chemistry and tissue examination, respectively.

### 2.6 Histopathological examination

The laboratory and clinical examinations were performed as previously described (Ahn et al., 2016). For histopathological examination, hepatic tissue sections were routinely processed for paraffin embedding and stained with hematoxylin and eosin. Histological evaluation was based on the modified NAFLD activity score (NAS) histological feature scoring system for liver lesions: steatosis (0–3), lobular inflammation (0–2), and hepatocellular ballooning (0–2) (Zhang et al., 2018).

Sirius red (Sigma‐Aldrich) staining was used for fibrillary collagen. Semiquantitative analysis of the Sirius red‐stained area (*n* = 5 animals per group) was performed using ImageJ software (NIH). Liver fibrosis was scored on a scale from 0 to 6 using the Ishak staging system as follows: 0, no fibrosis; 1, fibrous expansion of some portal areas, with or without short fibrous septa; 2, fibrous expansion of most portal areas, with or without short fibrous septa; 3, fibrous expansion of most portal areas with occasional portal‐to‐portal (P‐P) bridging; 4, fibrous expansion of portal areas with marked P‐P bridging as well as portal‐to‐central (P‐C) bridging; 5, marked P‐P and/or P‐C bridging with occasional nodules (incomplete cirrhosis); and 6, cirrhosis (Ishak et al., 1995).

### 2.7 Serological analysis

Blood samples were collected in order to determine the extent of liver damage, the serum levels of alanine aminotransferase (ALT), aspartate aminotransferase (AST), alkaline phosphatase (ALP), total cholesterol (TCHO), and TG were measured by Chemon Co., Ltd.

### 2.8 Quantitative real‐time PCR

Purified RNA from liver tissues was synthesized into cDNA using 5× First Strand cDNA Synthesis Master Mix (CellSafe) according to the manufacturer's protocol. The primer sequences are summarized in Table 1. PCR reactions were performed using the Mic Real‐Time PCR Cycler (Bio Molecular Systems). The relative expression levels were normalized to that of glyceraldehyde‐3‐phosphate dehydrogenase (GAPDH) using the 2^−ΔΔCT^ method.

**Table 1:** Primer sequences used in the present study

Primer	Sequence
αSMA	
Sense	5′‐GTCCCAGACATCAGGGAGTAA‐3′
Antisense	5′‐TCGGATACTTCAGCGTCAGGA‐3′
ColIA1	
Sense	5′‐GCTCCTCTTAGGGGCCACT‐3′
Antisense	5′‐CCACGTCTCACCATTGGGG‐3′
GAPDH	
Forward	5′‐ATG ATT CTA CCC ACG GCA AG‐3′
Reverse	5′‐CTG GAA GAT GGT GAT GGG TT‐3′
iNOS	
Sense	5′‐GAC CAG ATA AGG GCA AGC AC‐3′
Antisense	5′‐CTT GTC TTT GAC CCA GTA GC‐3′
TGFβ1	
Sense	5′‐CTCCCGTGGCTTCTAGTGC‐3′
Antisense	5′‐GCCTTAGTTTGGACAGGATCTG‐3′

### 2.9 Western blot analysis

For the immunoblot assay, supernatants (40 μg protein) were electrophoresed and immunoblotted onto a nitrocellulose membrane (Schleicher and Schuell). Proteins on the membrane were immunodetected using specific primary antibodies for 2 hr (Table 2). Bound membranes were detected using a chemiluminescent substrate (Miracle‐Star; iNtRON Biotech). After imaging, the density was analyzed by ImageJ software. The β‐actin antibody was used as the internal control.

**Table 2:** Primary antibodies used in the present study

Antigen	Species, antibody type, manufacturer	Dilution
Adipocyte protein (ap) 2	Goat, polyclonal, Santa Cruz Biotechnology	1:1,000
CCAAT/enhancer‐binding protein (C/EBP)α	Rabbit, polyclonal, Santa Cruz Biotechnology	1:1,000
C/EBPβ	Rabbit, polyclonal, Cell Signaling Technology	1:1,000
Fatty acid synthase (FAS)	Mouse, monoclonal, abcam	1:1,000
Ionized calcium‐binding adaptor molecule‐1 (Iba‐1)	Rabbit, polyclonal, Wako Pure Chemical Industries,	1:800
Peroxisome proliferator‐activated receptor γ (PPARγ)	Rabbit, polyclonal, Cell Signaling Technology	1:1,000
Sterol regulatory element‐binding protein (SREBP) 1c	Mouse, monoclonal, BD Bioscience	1:1,000
β‐actin	Mouse, monoclonal, Sigma‐Aldrich	1:10,000

### 2.10 Immunohistochemistry

Immunohistochemistry was performed using the ABC Elite kit (Vector Labs). The rabbit anti‐ionized calcium‐binding adapter molecule 1 (Iba‐1) was incubated with the liver sections. The peroxidase reaction was visualized using a diaminobenzidine substrate kit (Vector Labs). The slides were counterstained with hematoxylin before mounting. Quantitative analyses of Iba‐1‐immunostained areas (*n* = 5 animals per group) were performed with the aid of ImageJ software (NIH).

### 2.11 Statistical analysis

The results are presented as the mean ± standard error where in all cases level of statistical significance was considered as *p* < .05. For statistical analysis, the data were performed using a one‐way analysis of variance followed by the Student‐Newman‐Keuls post hoc test.

## 3 RESULTS

### 3.1 FBR diminished accumulation of lipid in 3T3‐L1 adipocytes

**Figure 2:**
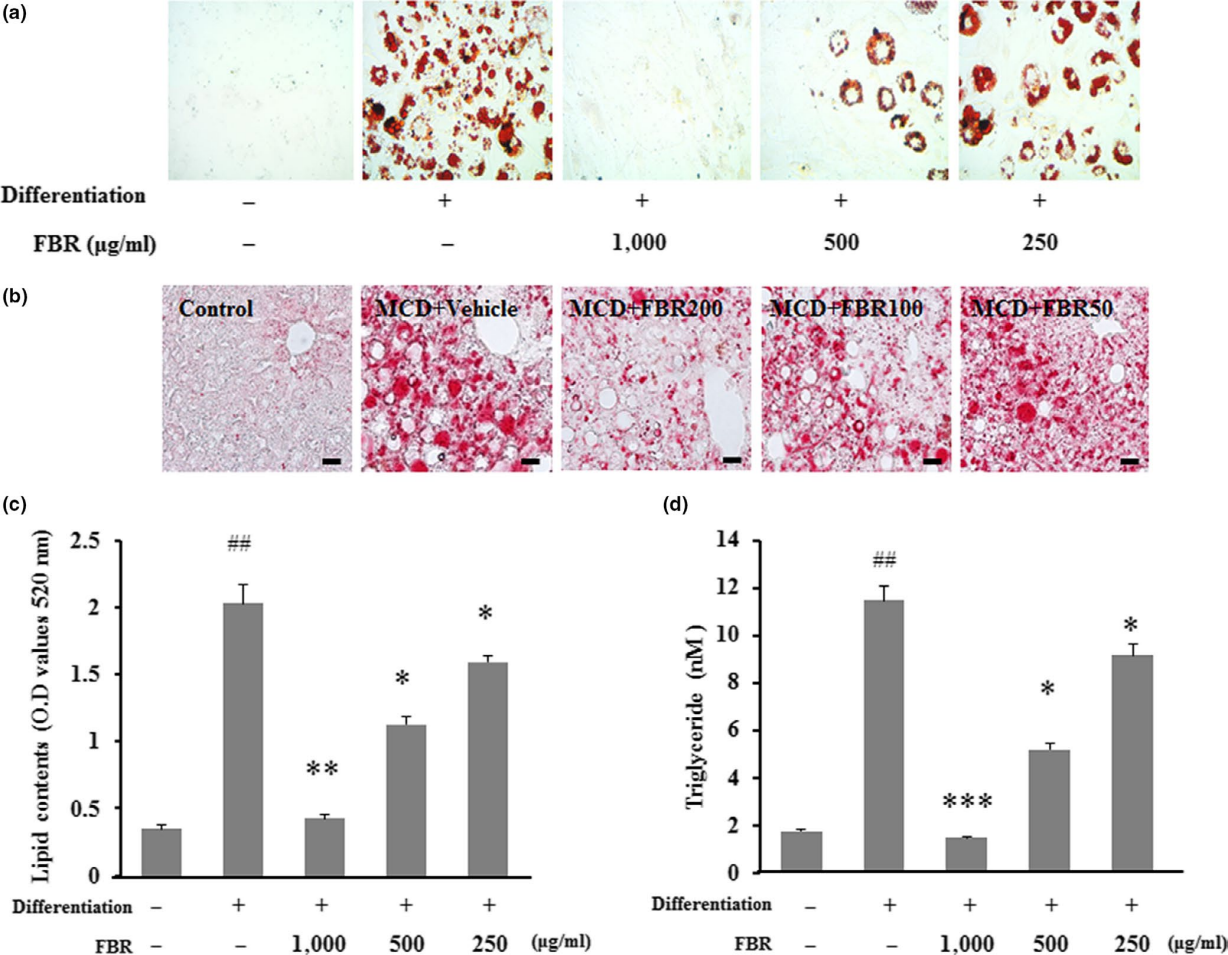
Intracellular lipid droplets were stained with Oil Red O and visualized by microscopy in 3T3‐L1 cells (a) and liver tissues (b). Bar graphs show the semiquantitative analysis of lipid droplets (c). Triglyceride contents were quantified using individual kits (d). Data are presented as the mean ± standard error (*SE*). *^#^p* < .05, *^##^p* < .01 versus control cells; *p < .05, ***p < .001 versus vehicle‐treated cells

Differentiated 3T3‐L1 adipocytes were visualized by Oil Red O staining, to evaluate the effects of FBR on the intracellular lipid storage (Figure 2a). Fermented black radish significantly reduced the amount of lipid in 3T3‐L1 adipocytes in a dose‐dependent manner (*p* < .05 and *p* < .01 vs. nonFBR‐treated control cells for 250 and 500 μg/ml and 1,000 μg/ml, respectively; Figure 2a,c). Similar results were observed in MCD diet‐induced mouse livers (Figure 2b). This noted reduction of lipid accumulation was further confirmed with the TG quantification assays. As illustrated in Figure 1c, compared to the FBR‐untreated control cells, treatment with FBR significantly decreased the TG content in 3T3‐L1 adipocytes (*p* < .05 for 250 and 500 μg/ml and *p* < .01 for 1,000 μg/ml; Figure 2d). These findings suggest that FBR treatment reduced lipid accumulation in 3T3‐L1 adipocytes and liver tissues.

### 3.2 FBR downregulates the expression of proteins linked with adipogenesis in 3T3‐L1 adipocytes

**Figure 3:**
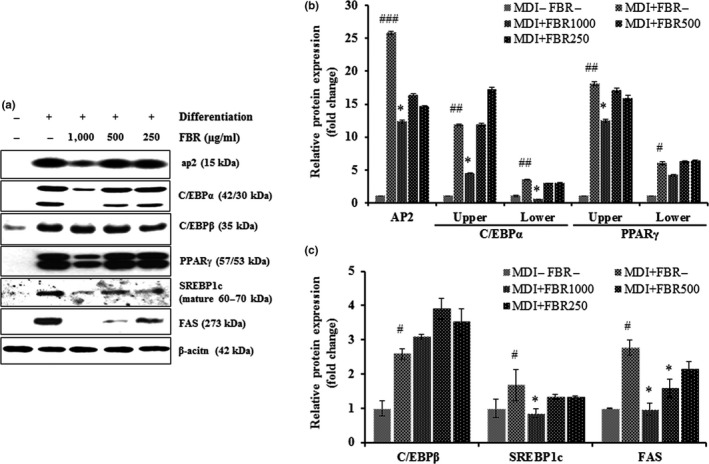
Representative immunoblots of ap2, C/EBPα, C/EBPβ, PPARγ, SREBP1c, FAS, and β‐actin expression (a), and expression of ap2, C/EBPα, PPARγ, SREBP1c, and FAS relative to β‐actin level in FBR‐treated 3T3‐L1 cells (b and c). Data are presented as the mean ± *SE*. *n* = 3 per group. *^#^p* < .05, *^##^p* < .01, *^###^p* < .001 versus control cells; *p < .05 versus vehicle‐treated cells

To understand the molecular mechanisms underlying the antiadipogenic effects of FBR, we analyzed the expression levels of adipogenic genes involved in lipid uptake and metabolism, including ap2, C/EBPα, C/EBPβ, PPARγ, SREBP1c, and FAS. All genes were expressed at significantly higher levels in differentiated control cells than in undifferentiated control cells (*p* < .05). However, the expression levels of ap2, C/EBPα, PPARγ, SREBP1c, and FAS were significantly reduced in cells treated with 1,000 or 500 µg/ml FBR (*p* < .05). A significant difference of the expression levels of C/EBPβ was not detected between FBR‐treated cells and untreated cells (Figure 3).

### 3.3 Evaluation of biochemical indicators in serum

To verify the protective role of FBR during MCD diet‐induced hepatic injury, biochemical analyses of serum enzymes were performed as shown in Table 3. The serum indicators of hepatocellular damage, namely, ALT, AST, and ALP were higher significantly in the vehicle‐treated group than in the control group (*p* < .01). However, the activity of serum ALT was pronouncedly reduced in the 100, 200, and 400 mg/kg FBR‐treated groups (*p* < .05 and *p* < .01, respectively). Furthermore, the activities of serum AST and ALP were significantly reduced in the 200 mg/kg FBR‐treated group (*p* < .05) compared with those in the vehicle‐treated group, as well as those in the silymarin‐treated group (Table 3). Levels of serum lipids, including TCHO and TG, were significantly higher in the vehicle‐treated group than in the normal control group (*p* < .01 and *p* < .05, respectively). Serum TG levels were significantly reduced in the 100, 200, and 400 mg/kg FBR‐treated groups compared with those in the vehicle‐treated group (*p* < .01). However, the levels of serum TCHO did not significantly differ among the FBR‐treated groups. These results imply the potentiality of FBR treatment in liver protection against MCD diet‐induced hepatic injury.

**Table 3:** Biochemical analysis of sera from MCD diet‐induced NALFD mice with or without FBR treatment

Treatment	ALT (U/L)	AST (U/L)	ALP (U/L)	TCHO (mg/dl)	TG (mg/dl)
Normal control	84.3 ± 2.8	16.8 ± 0.3	63.8 ± 6.4	75.6 ± 3.3	45.8 ± 3.3
MCD diet + Vehicle	528.4 ± 37.9###	447.2 ± 178.7###	129.0 ± 16.0##	34.4 ± 10.9##	65.8 ± 7.2#
MCD diet + FBR (50 mg/kg)	562.2 ± 57.8###	407.0 ± 57.7###	133.8 ± 11.7##	29.5 ± 1.0	62.5 ± 8.2
MCD diet + FBR (100 mg/kg)	334.3 ± 11.9###, *	299.3 ± 31.5##	118.0 ± 8.2#	18.4 ± 1.9	30.8 ± 2.1##, **
MCD diet + FBR (200 mg/kg)	248.7 ± 4.6##, **	238.9 ± 30.6##, *	88.8 ± 13.3#, *	25.6 ± 3.8	25.4 ± 6.5##, **
MCD diet + FBR (400 mg/kg)	360.8 ± 37.3##, *	377.3 ± 71.4##	103.6 ± 3.5#	27.2 ± 3.2	30.0 ± 3.9##, **
MCD diet + Silymarin (100 mg/kg)	309.6 ± 13.4##, *	193.2 ± 33.9##, *	86.0 ± 8.04#, *	22.6 ± 1.0	22.2 ± 1.7##, **

Note

Each value is represented as the mean ± *SE*.

^#^ *p* < .05,

^##^ *p* < .01,

^###^ *p* < .001 versus normal controls.

* *p* < .05,

** *p* < .01 versus MCD diet‐induced NALFD with vehicle treatment group.

### 3.4 FBR improved the histopathological changes in the liver

**Figure 4:**
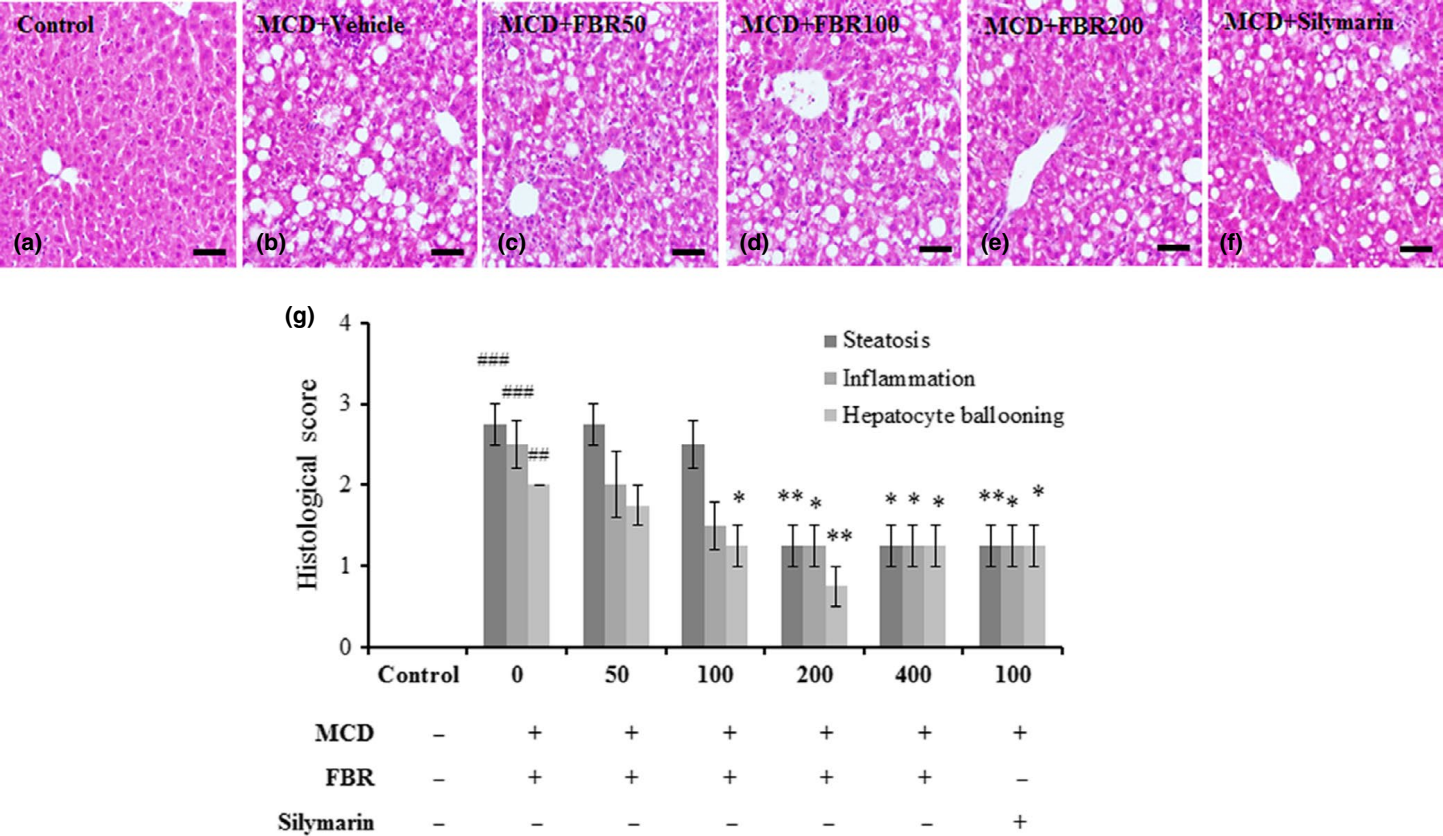
Each panel shows a photograph from each group, representatively (a‐f). The histological characteristics of NAFLD were assessed in each group by three blind observers using hematoxylin and eosin staining (g). Data are presented as the mean ± *SE*. ^##^ *p* < .01, ^###^ *p* < .001 versus normal controls; *p < .05, **p < .01 versus vehicle‐treated group. Scale bars in a–f represent 20 μm

There is a evidence from histopathological studies that FBR protects against MCD diet‐induced liver injury (Figure 4). In the normal control group (Figure 4a), histological sections showed hepatocytes with well‐preserved cytoplasm, prominent nuclei, and central veins. The livers of MCD diet‐induced mice exhibited moderate steatohepatitis (Figure 4b). Conversely, administration of FBR with the MCD diet prevented rather than increased liver injury induced by MCD diet alone, in a dose‐dependent manner (Figure 4c–f). Additionally, we performed pathophysiological evaluation using the NAFLD activity scoring system (Zhang et al., 2018). The results suggest that pretreatment with 200 and 400 mg/kg FBR significantly inhibited steatosis, hepatocyte ballooning, and inflammation compared with vehicle treatment (*p* < .05 and *p* < .01, respectively). Similar effects were observed in the silymarin‐treated group (Figure 4g).

### 3.5 FBR treatment inhibited inflammation in the liver

**Figure 5:**
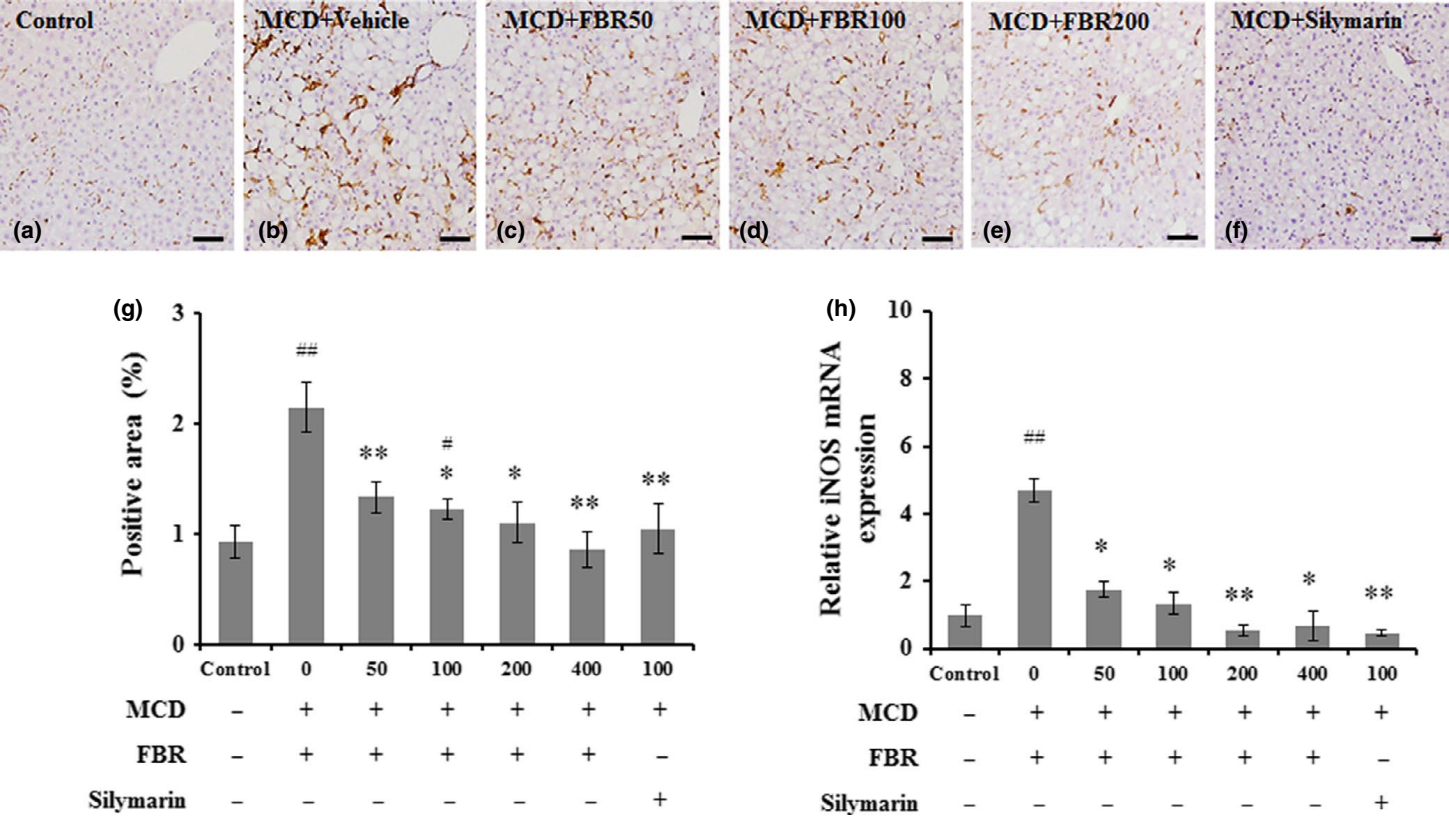
Immunohistochemical staining of Iba‐1 in liver sections (a‐f). Bar graph shows the semiquantitative analysis of Iba‐1‐positive area (g) and real‐time PCR analysis of mRNA levels of hepatic iNOS (*n* = 5 per group) (h). Scale bars in (a–f) = 50 μm. Values in (g and h) are means ± *SE*. ^#^ *p* < .05, ^##^ *p* < .01 versus normal controls; *p < .05, **p < .01 versus vehicle‐treated group

Liver injury is importantly indicated by the activation of Kupffer cells/macrophages (Ahn et al., 2016). Iba‐1‐positive Kupffer cells were detected along the sinusoids of mouse livers in the normal control group (Figure 5a), in which no infiltration of inflammatory cells was observed. Activation of Kupffer cells and infiltration of inflammatory cells were detected in the livers of MCD diet‐induced mice (Figure 5b), whereas pretreatment with FBR (Figure 5c‐e) and silymarin (100 mg/kg; Figure 5f) reduced the numbers of Iba1‐positive cells. Furthermore, the percentage of Iba‐1‐positive areas in the vehicle‐treated group was significantly greater than that in the normal control group (*p* < .01). However, these areas were significantly reduced in the FBR treatment groups (50 and 400 mg/kg FBR, *p* < .01; 100 and 200 mg/kg FBR, *p* < .05) and silymarin‐treated groups (*p* < .01; Figure 5g). Hepatic iNOS mRNA levels increased significantly in the vehicle‐treated group than that of the control group (*p* < .01). However, these effects were reversed in the FBR treatment groups (50, 100, and 400 mg/kg FBR, *p* < .05; 200 mg/kg FBR, *p* < .01), as well as in the silymarin‐treated group (Figure 5h).

### 3.6 FBR prevented hepatic fibrosis in the liver

**Figure 6:**
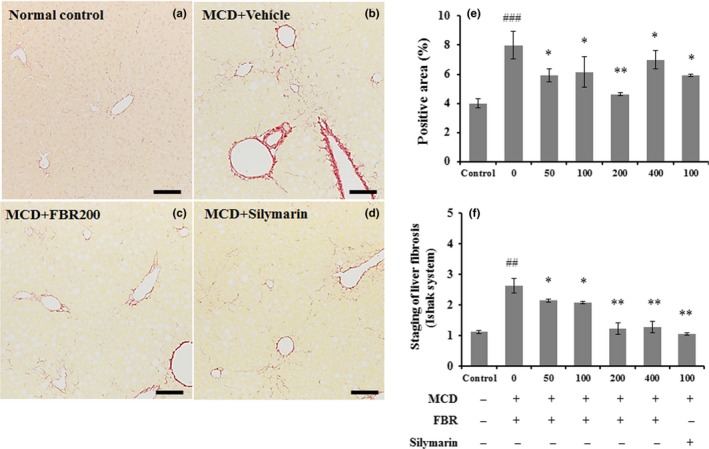
Sirius red staining was applied to evaluate hepatic collagen deposition (a–d). Bar graph shows the semiquantitative analysis of Sirius red‐positive area (e) and Ishak staining system (f) in each group. Data are presented as the mean ± *SE* (*n* = 5 per group). #*p* < .05, ##*p* < .01, ###*p* < .001 versus normal controls; *p < .05, **p < .01 versus vehicle‐treated group. Scale bars in a–d represent 100 μm

**Figure 7:**
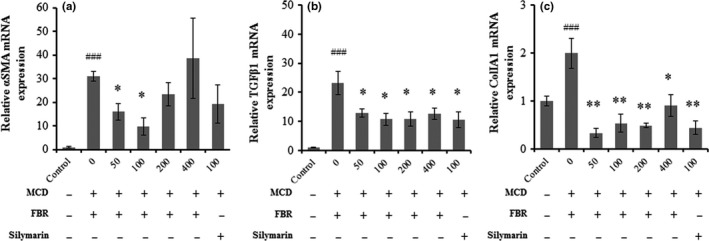
Liver mRNA expression of αSMA (a), TGFβ1 (b), and Col1A1 (c). Data are presented as the mean ± *SE*. *n* = 5 per group. ###*p* < .001 versus normal controls; *p < .05, **p < .01 versus vehicle‐treated group

In order for determining that FBR pretreatment alleviate hepatic fibrosis, liver sections were stained with Sirius red stain. Collagen fibers were detected around perivenular regions of the normal control (Figure 6a). However, collagen tissue proliferation by fibrosis was detected in the vehicle‐treated group (Figure 6b). Fermented black radish and silymarin treatment significantly reduced collagen tissue proliferation in the liver (Figure 6c,d) compared with the vehicle‐treated group. The positive area with Sirius red staining was significantly higher in the vehicle‐treated group (7.99 ± 0.3%, *p* < .001) than that in the normal control group (3.99 ± 0.31%). The groups treated with 50 mg/kg (5.91 ± 0.94%, *p* < .05), 100 mg/kg (6.15 ± 1.06%, *p* < .05), 200 mg/kg (4.63 ± 0.09%, *p* < .01), and 400 mg/kg FBR (6.99 ± 0.62%, *p* < .05), and 100 mg/kg silymarin (5.91 ± 0.08%, *p* < .05) showed significantly lower percent areas of collagen tissue compared with the liver tissue in the vehicle‐treated group (Figure 6e). Ishak histological scores were significantly higher in the vehicle‐treated group (2.62 ± 0.24, *p* < .01) than in the control group (1.11 ± 0.05). In the FBR treatment groups, the MCD diet‐induced increase in fibrosis score was counteracted in a dose‐dependent manner (*p* < .05 for 50 and 100 mg/kg and *p* < .01 for 200 and 400 mg/kg), as well as in the silymarin‐treated group (*p* < .01; Figure 6f). Consistently, in the MCD diet‐induced hepatic injury lead elevated liver mRNA expression of the profibrogenic genes such as α‐smooth muscle actin (SMA), transforming growth factor beta‐1 (TGFβ1), and collagen type I alpha1 chain (Col1A1) while dramatic reduction of the levels was observed by FBR treatment (Figure 7a–c).

## 4 DISCUSSION

This is the first report of the hepatoprotective effects of oral uptake of FBR in the chronic NAFLD model. We determined that FBR had a hepatoprotective effect on differentiated 3T3‐L1 cells and the MCD diet‐fed mice, a well‐established nutritional model of NAFLD (Lee et al., 2018). Our results showed that FBR attenuated adipocyte differentiation and lipid accumulation in 3T3‐L1 preadipocytes and suppressed key regulators of hepatic steatosis, inflammation, and fibrosis in the livers of animal model. These current findings are compatible with the previously reported hepatoprotective effects of FBR on carbon tetrachloride‐induced acute hepatic injury in rats (Kim et al., 2017).

Regarding the mechanism of the hepatoprotective effects of FBR, we postulate that FBR‐induced reductions in lipid accumulation and TG content in 3T3‐L1 cells are related to the reduction in adipogenic transcription factors and the expression of genes related to lipid accumulation. It has been reported that inhibition of SREBP1c mediated lipogenesis and activation of PPAR‐mediated oxidation of free fatty acids are two principal molecular targets in the treatment of nonalcoholic steatohepatitis (Musso, Cassader, & Gambino, 2016), because SREBP1c is known to play a vital role in hepatic accumulation of TG and is a major regulator of genes involving lipogenesis in the liver such as ACC and FAS (Shimano, 2009).

The current study further revealed that FBR reduces the hepatic lipid accumulation along with the downregulation of genes related to adipogenic transcription factors in 3T3‐L1 cells, including C/EBPα, SREBP 1c, and PPARγ; and to genes related to lipid accumulation, ap2, and FAS.

The in vivo mouse study revealed that FBR and silymarin treatment significantly ameliorated MCD diet‐induced liver damage, as depicted with decreased levels of liver dysfunction enzymes (ALT, AST, and ALP) and decreased serum lipid content activity. Early‐stage of progressive of nonalcoholic steatohepatitis is characterized with increased TG and TCHO accumulation in the liver that results from impaired of fatty acid oxidation and amplified fatty acid synthesis (Banini & Sanyal, 2016). Our results showed that FBR suppressed the increased levels of liver dysfunction enzymes and serum lipid content. These findings suggest that FBR ameliorates hepatic steatosis through modulating the crucial regulators involved in lipid metabolism in a manner similar to silymarin treatment.

We hypothesized the usefulness of FBR in preventing various types of hepatic damage induced by inflammation in addition to the injury caused by lipid accumulation. Based on histopathological examination, we found that FBR treatment considerably ameliorated MCD diet‐induced hepatic changes and hepatotoxicity, including steatosis, inflammation, hepatic ballooning, and fatty degeneration. This is in accordant with our previous reports on the hepatoprotective effects of black radish extract (Ahn et al., 2018).

Hardly any studies have investigated on the anti‐inflammatory effect of FBR on liner using the MCD diet‐induced chronic injury model. Activation of macrophages and Kupffer cells in liver injury is a well‐known phenomenon (Ahn et al., 2016). Our results showed that FBR treatment suppressed macrophage infiltration and resulted in increased levels of pro‐inflammatory mediators such as iNOS mRNA levels in the mouse liver, suggesting that the hepatoprotective role of FBR is associated with anti‐inflammatory effects as well as decreased activation of Kupffer cells/macrophages. Our results support previous findings that FBR alleviates hepatic inflammation in MCD diet‐induced steatohepatitis.

Various persistent liver injuries end up with hepatic fibrosis. This common pathological condition is featured by deposition of extracellular matrix in the perisinusoidal space due to disparity between the synthesis and the degradation of extracellular matrix (Liu et al., 2015). In fact, an effective treatment strategy should address the prevention or reversal of the process leading to hepatic fibrosis (Chen et al., 2013). In our study, the hepatic fibrosis induced by the MCD diet and the hepatoprotective effects of FBR were clear based on histological findings. According to the current findings, FBR treatment alleviated the progression of MCD diet‐induced chronic liver fibrosis in mice as explained with the fibrosis scores and hepatic collagen content. Moreover, the amelioration of hepatic fibrosis by FBR treatment was partly mediated by hepatic mRNA expression levels of fibrosis‐related genes including αSMA, TGFβ1, and Col1A1.

## 5 CONCLUSIONS

**Figure 8:**
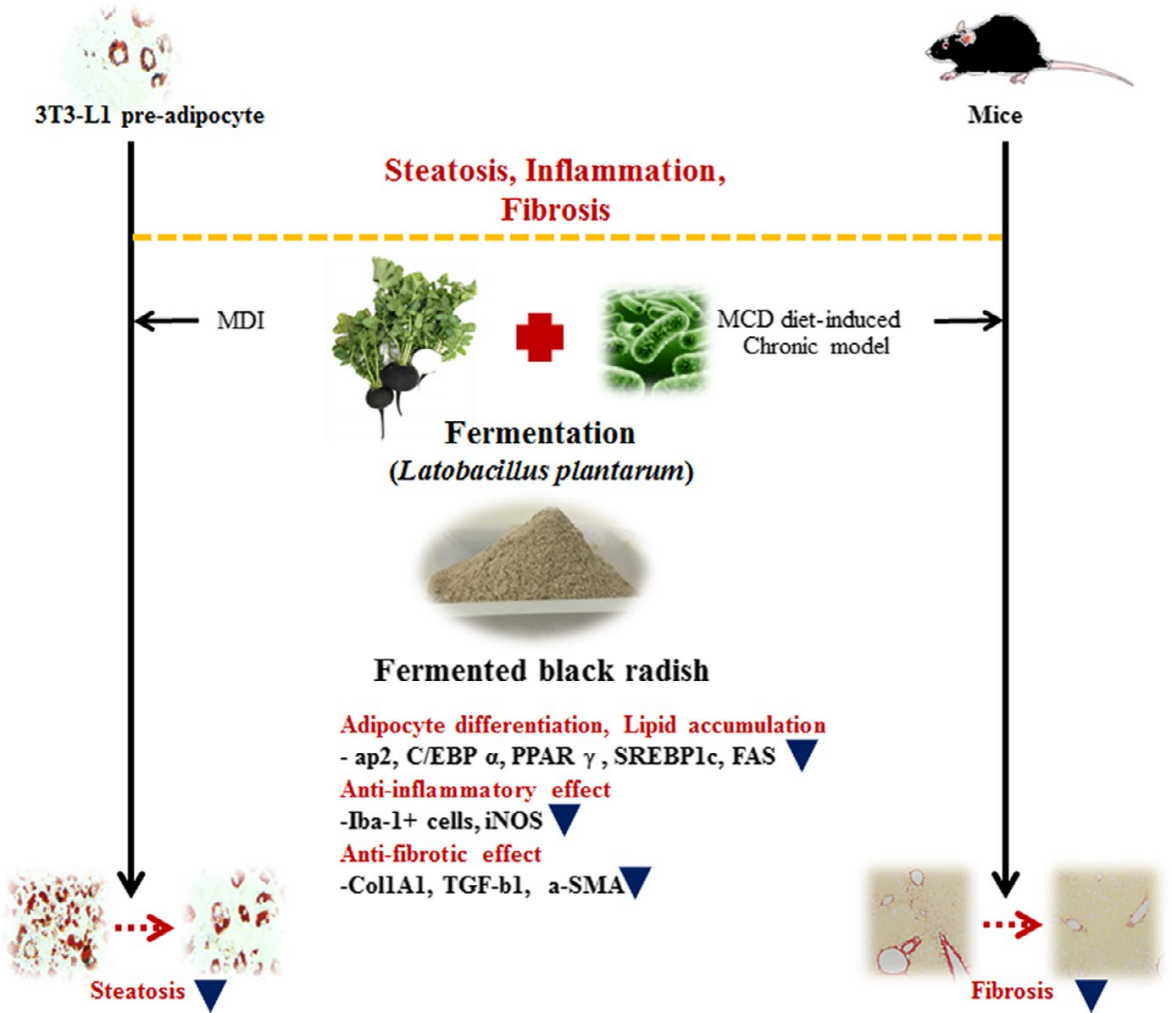
Schematic diagram of the proposed molecular effects of FBR in in vitro and in vivo

In conclusion, FBR suppressed hepatic lipid accumulation, inflammation, and fibrosis by downregulating adipogenic transcription factors and genes associated with lipid accumulation, inflammation, and fibrosis. Overall, the hepatoprotective effects of FBR in the MCD diet‐induced liver injury model suggest its potential therapeutic use in NAFLD (Figure 8).

## 6 ACKNOWLEDMENTS

This work was supported by Korea Institute of Planning and Evaluation for Technology in Food, Agriculture, Forestry and Fisheries (IPET) through Agri‐Bio industry Technology Development Program, funded by Ministry of Agriculture, Food and Rural Affairs (MAFRA) (Grant number: 316006‐05‐1‐HD040).

## CONFLICT OF INTEREST

The authors declare that they do not have any conflict of interests.

## ETHICAL APPROVAL

This study does not involve any human or animal testing.
